# High protective efficacy of rice bran against human rotavirus diarrhea via enhancing probiotic growth, gut barrier function, and innate immunity

**DOI:** 10.1038/srep15004

**Published:** 2015-10-13

**Authors:** Xingdong Yang, Erica Twitchell, Guohua Li, Ke Wen, Mariah Weiss, Jacob Kocher, Shaohua Lei, Ashwin Ramesh, Elizabeth P. Ryan, Lijuan Yuan

**Affiliations:** 1 Department of Biomedical Sciences and Pathobiology, Virginia-Maryland College of Veterinary Medicine, Virginia Polytechnic Institute and State University, Blacksburg, Virginia, USA; 2 Department of Environmental and Radiological Health Sciences, College of Veterinary Medicine and Biomedical Sciences, Colorado State University, Fort Collins, Colorado, USA.

## Abstract

Previously, we showed that rice bran (RB) was able to reduce human rotavirus (HRV) diarrhea in gnotobiotic pigs. Here, we investigated its effect on the growth of diarrhea-reducing probiotic *Lactobacillus rhamnosus GG* (LGG) and *Escherichia coli* Nissle (EcN), and the resulting effects on HRV diarrhea, gut epithelial health, permeability and innate immune responses during virulent HRV challenge. On 3, 5, and 7 days of age pigs were inoculated with 2 × 10^4^ colony-forming-units LGG+EcN to initiate colonization. Daily RB supplementation (replacing 10% calorie intake) was started at 5 days of age and continued until euthanasia. A subset of pigs in each group was challenged orally with 10^5^ focus-forming-units of virulent HRV at 33 days of age. RB completely prevented HRV diarrhea in LGG+EcN colonized pigs. RB significantly promoted the growth of both probiotic strains in the gut (~5 logs) and increased the body-weight-gain at 4–5 weeks of age compared to non-RB group. After HRV challenge, RB-fed pigs had significantly lower ileal mitotic index and villus width, and significantly increased intestinal IFN-γ and total IgA levels compared to non-RB group. Therefore, RB plus LGG+EcN colonization may represent a highly effective therapeutic approach against HRV and potentially a variety of other diarrhea-inducing enteric pathogens.

Human rotavirus (HRV) is a segmented, double-stranded RNA virus in the *Reoviridae* family. It is a leading cause of severe gastroenteritis among children less than 5 years old, resulting in 2 million hospitalizations and 450,000 deaths each year, mainly in developing countries^1^. Currently, there are two commercially available vaccines for HRV, Rotarix (RV1, GlaxoSmithKline, FDA approval in 2008) and RotaTeq (RV5, Merck, FDA approval in 2006). Although both vaccines have contributed to significant reduction in rotavirus diarrhea incidences in young children since their introduction, efficacy varies by geographical regions and national economic status (>90% to 29%), with lower rate of protection seen in developing countries^2^. Animal models have been essential in the development of rotavirus prevention and therapeutic strategies, and particularly with the gnotobiotic (Gn) pig model of HRV infection and diarrhea. The neonatal Gn pig has high translational relevance for study of the gastrointestinal tract in young children and has been widely used for rotavirus infection and immunity^3^,^4^, vaccines^5^,^6^,^7^ and vaccine adjuvants^8^,^9^.

Rice bran contains prebiotic compounds^10^ and a variety of bioactive components (i.e. polyphenols, fatty acids and peptides)^11^,^12^ that have been shown to have promising protective effects against diseases such as cancer^13^, obesity^14^ and diabetes^15^ and immune modulatory effects^16^. Its therapeutic effects against enteric pathogen infections and diseases have also been studied in animal models^17^,^18^. By feeding mice a 10% RB diet, it was shown that RB reduced the colonization and invasion of *Salmonella enterica* serovar typhimurium into enterocytes and intestinal mucosa^17^. MGN-3, an arabinoxylan extracted from RB, was shown to inhibit human immunodeficiency virus (HIV) replication in primary cultures of peripheral blood mononuclear cells through directly inhibiting HIV entry and replication^19^. In another mouse study, 10% dietary RB feeding for 28 days resulted in increased production of mucosal and systemic IgA^20^. In these mouse studies, modulation of gut microbiota, such as 170-fold increase in the population of probiotic *Lactobacillus* spp. and decreased colonization of mucin degrading microbes (phylum Verrucomicrobia), was proposed as one of the possible mechanisms for RB to reduce the colonization and invasion of *Salmonella* bacteria^17^. We previously demonstrated that dietary RB feeding (10% RB diet) for 28 days significantly reduced HRV induced diarrhea, without decreasing HRV replication and shedding in Gn pigs^18^. Therefore, RB or its components protect against enteric pathogen infections and diseases probably through multiple mechanisms, including direct anti-microbial activities, prebiotic effects, and promoting intestinal epithelial health and mucosal immune responses. Identifying the RB mediated gut barrier and mucosal immune mechanisms involved was the goal of the current study.

*Lactobacillus rhamnosus* GG (LGG) is a gram-positive bacterium in the *L. rhamnosus* species that was first isolated in 1983 by Barry R. Goldin and Sherwood L. Gorbach^21^. It is widely studied for treatment and prevention of gastrointestinal diseases and infections, and increasingly for extra-intestinal diseases as well, such as atopic dermatitis, allergic reactions, urogenital tract infections, and respiratory tract pathogens^22^. It has been shown to reduce the severity and duration of rotavirus diarrhea and persistent diarrhea in multiple clinical trials^23^,^24^. LGG has also been found to reduce intestinal permeability in children with irritable bowel syndrome^25^. *Escherichia coli* Nissle 1917 (EcN) is one of the best characterized probiotics used to reduce acute and protracted diarrhea^26^, and was shown to protect Gn pigs from lethal challenge by *Salmonella* typhimurium. Given the above discussed effects of RB, LGG and EcN individually on rotavirus and *Salmonella* infection and diarrhea, studies to examine their combined therapeutic effects in the Gn pig model are warranted.

In this study, we hypothesized that RB can promote the growth of LGG and/or EcN, enhance gut health, reduce gut permeability, and together with LGG and EcN colonization, provide effective protection against HRV diarrhea and shedding. The objectives of this study were to identify: 1) the prebiotic effect of dietary RB on the growth of both LGG and EcN strains; 2) the protective effect of dietary RB supplementation against HRV diarrhea and shedding in LGG and EcN colonized Gn pigs; and 3) the effects of RB on intestinal health, permeability and innate immunity in LGG and EcN colonized Gn pigs.

## Materials and Methods

### Experimental groups

Near-term pigs (Large White crossbred) were derived by hysterectomy and maintained in germ-free isolator units as described^27^. Weekly sterility checks were conducted by collecting rectal swabs and plating on blood agar plates and in thioglycollate media. Neonatal Gn pigs were randomly divided into four treatment groups: probiotics plus RB feeding (RB+LGG+EcN), RB only (RB only), probiotics only (LGG+EcN), and mock control (mock). *Lactobacillus rhamnosus* GG strain (ATCC 53103) and EcN (a gift from Dr. Jun Sun, Rush University, Chicago, IL) used in the current study were propagated in Lactobacilli MRS broth (Weber, Hamilton, NJ) and Luria Broth (LB) media, respectively. LGG and EcN 1:1 mixture at 10^4^ CFU/dose each were administered orally via a needle-free syringe to pigs at post-partum day (PPD) 3, 5 and 7 to initiate colonization. The low dosage is purposely selected to be well below the therapeutic doses (10^9^ to 10^12^ CFU). Heat-stabilized, gamma irradiated RB (Calrose variety) was added to the Gn pigs’ milk diet (ultra-high-temperature treated cow-milk) replacing 10% daily calorie intake, starting at PPD 5 until the end of experiment. A subset of pigs from RB+LGG+EcN and LGG+EcN groups were euthanized before challenge on PPD 33 (n = 6). The rest of the pigs were challenged orally with 10^5^ focus-forming units (FFU) of the virulent Wa strain (G1P1A[8]) HRV on PPD 33 (PCD 0) and euthanized on post-challenge day (PCD) 3 (n = 6) or PCD 7 (n = 6). The pigs were weighed weekly starting on PPD 5 until euthanasia. Rectal swabs were collected daily for monitoring of virus shedding by ELISA and CCIF from PCD 0 to 7. Rectal swabs were also collected daily for monitoring of diarrhea from PCD 0 to 7. Diarrhea scoring was conducted by different researchers independently and are defined as 0) solid, 1) pasty, 2) semi-liquid, and 3) liquid. Scores of 2 or higher are considered diarrheic. All animal experiments were conducted according to the protocols approved by the Institutional Animal Care and Use Committee at Virginia Polytechnic Institute and State University.

### Titration of HRV antigen and infectious virus shedding

To determine the effects of dietary RB supplementation and LGG and EcN colonization on HRV shedding, rectal swabs were collected daily upon virulent HRV challenge from PCD 0 to 7. Rectal swabs were processed by rising two swabs in 8 ml Diluent #5 (MEM, 1% penicillin and streptomycin, 1% HEPES) and then centrifuged at 2100 rpm for 15 minutes at 4 °C. Supernatants were then aliquoted and stored at −20 °C. Viral antigen and infectious viruses in the rectal swabs were then measured using ELISA and CCIF, respectively as previously described^28^. Rectal swabs from mock challenged Gn pigs were used as negative controls.

### LGG and EcN counting

Rectal swabs from the LGG+EcN colonized groups were collected on PPD 5, 10, 15, 26 and 33 for LGG and EcN counting. LGG was cultured anaerobically using BBL Gaspak jars (Fisher, Hanover Park, IL, USA) containing Anaerogen packs (BD) according to a protocol in a previous publication^29^. For titration of LGG shedding, one fecal swab is washed in 4 ml 0.1% peptone water (10-fold dilution) and 10^2^, 10^3^, and 10^4^ fold dilution series were then prepared. Then 0.1 ml of each dilution series was spread on a MRS agar plates and incubated anaerobically in a 37 °C incubator for 24 hours. Plates with 20–200 bacteria colonies were then enumerated. The results are expressed as CFU/ml. For EcN counting, basically the same procedures were followed as for LGG, except for the replacement of 0.1% peptone water and MRS with LB media and plates were cultured aerobically (placed directly in the 37 °C incubator).

### Ileum histopathology

After euthanasia, sections of ileum from the LGG+EcN colonized groups were collected for histopathologic examination. Samples were fixed in 4% paraformaldehyde, routinely processed for H&E staining and evaluated with light microscopy. The pathologist was blinded to identification of the animal until after microscopic analysis of all samples was complete. A histopathological scoring system for ileal samples was developed using guidance from previous publications^30^,^31^,^32^,^33^,^34^. The mitotic index was obtained by dividing the total number of mitotic figures in 50 randomly selected crypts not associated with Peyer’s patches by 50. Ten randomly selected ileal villi and crypts, not overlying Peyer’s patches were measured for each sample to provide an average villus length and crypt depth. Villus length was measured from the tip of the villus to the junction with the crypt and crypt depth was measured from the junction with the villus to crypt base. Villus length to crypt depth ratio (V:C) was obtained by dividing the mean villus length by the mean crypt depth for each sample. V:C score was assigned as follows: 0 (normal), > or = 6:1; 1 (mild), 5.0-5.9:1; 2 (moderate), 4.0-4.9:1; 3 (marked), 3-3.9:1; 4 (severe), <3:1. The mid-villus width of 10 random ileal villi, not overlying Peyer’s patches were measured and averaged. The number of cells within the ileal lamina propria was given a subjective score ranging from 1+ to 4+.

### Alpha-1-antitrypsin (A1AT) concentration

Alpha-1-antitrypsin is a marker of gut permeability. After euthanasia, large intestinal content (LIC) samples from the LGG+EcN colonized groups were collected in cryovials and immediately frozen in liquid nitrogen until permeability analysis (measuring A1AT level) using a commercial porcine A1AT ELISA kit (BIOTANG Inc. Lexington, MA). Briefly, LIC samples were thawed. Then 0.1 ml samples were diluted in 0.2 ml sample dilution buffer, mixed and centrifuged at 2000 rpm for 15 minutes at 4 °C. Supernatant was collected and 0.1 ml of each sample was added to duplicated wells on the plate. The ELISA was performed following exact instructions contained in the kit. OD values were measured at 405 nm within 30 minutes of adding stop solution. Final concentrations (μg/ml) were then obtained using the standard curve methods (polynomial with an order of 3).

### Intestinal IFN-γ concentration and total IgA and IgG titers

Small and large intestinal content (SIC and LIC) samples from the LGG+EcN colonized groups were collected upon euthanasia, and stored in −20 °C until analysis. IFN-γ concentration was measured using Swine IFN-γ VetSet™ ELISA Development Kit (Cat. No. VS0259S-002, Kingfisher, MN) according to the manufacturer’s instructions. Briefly, SIC and LIC samples were thawed and diluted 2-fold in sample dilution buffer provided in the kit. Diluted samples were added to the plate at 100 μl/well and incubated at room temperature (20–25 °C) for 1 h. After washing 4 times with washing buffer, swine IFN-γ detection antibody was added and incubated at room temperature for 1 hour. After washing, streptavidin-HPR working solution was then added and incubated for 30 min at room temperature. TMB substrate solution was added to plate at 100 μl/well after the same washing procedure. After incubation in the dark at room temperature for 20–30 min, reactions were stopped by adding 100 μl/well stop solution. Absorbance was read using Promega GloMax® Discover plate reader at 450 nm wavelength. Average concentrations were calculated from the duplicated wells for each sample. Total IgA and IgG titers in SIC and LIC were determined by ELISA as we previously described^18^.

### Statistical Analysis

Kruskal-Wallis rank sum test was used for comparisons of virus shedding duration and titer, diarrhea duration and score, body weight change, LGG and EcN count, ileum histology score, and A1AT concentration. Fisher’s exact test was used for comparisons of percentages of virus shedding and diarrhea. Statistical significance was assessed at P < 0.05.

## Results

### RB completely protected against rotavirus diarrhea in LGG and EcN colonized Gn pigs

The effects of RB on HRV induced diarrhea and virus shedding in LGG and EcN colonized Gn pigs were assessed (Table 1). RB alone significantly protected against HRV diarrhea (80% protection rate) but did not reduce HRV shedding (compared the RB only group with the Mock group). LGG+EcN alone also significantly reduced incidence of HRV diarrhea (50% protection rate), but significantly prolonged HRV shedding (compared the LGG+EcN group with the Mock group). RB+LGG+EcN completely protected against HRV diarrhea (100% protection rate); however, it did not significantly alter the percentage and duration of HRV shedding compared to the Mock group. When comparing the LGG+EcN and the RB+LGG+ EcN pigs, the latter had no diarrhea and significantly reduced HRV shedding, with significantly delayed onset (1.2 versus 2.8 days), shortened mean duration (6.8 versus 5.2 days) and ~217-fold reduction in peak virus titer (1.3 × 10^5^ versus 6 × 10^2^ FFU/ml). Thus, although RB or LGG+EcN alone did not reduce virus shedding, RB supplementation prevented the increase of HRV shedding (duration and peak titers) in the LGG+EcN colonized pigs.

### RB significantly enhanced the growth and colonization of LGG and EcN

**Figure 1:**
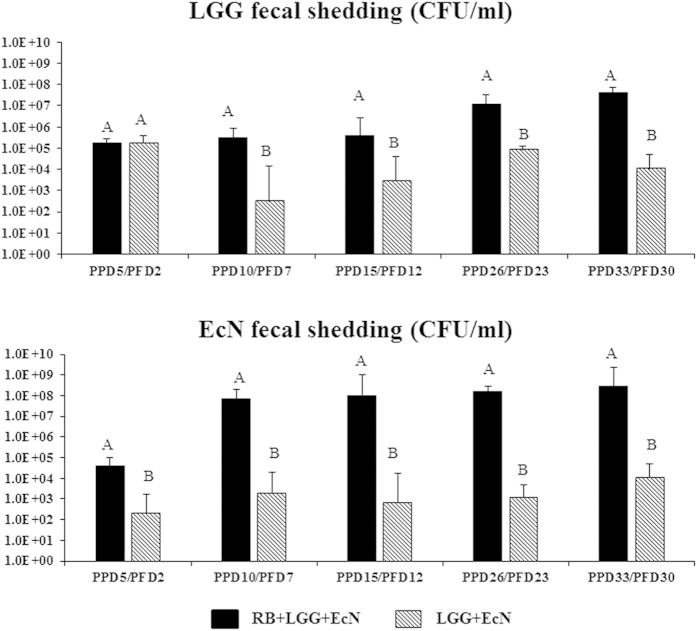
RB promoted the growth and colonization of the probiotics LGG and EcN in Gn pigs. Rectal swabs were washed in 4 ml 10% peptone water (10 fold dilution) and additional 10 fold series dilutions from 10^2^ to 10^4^ were prepared and plated on LGG agar plates (LGG counting) or LB agar plates (EcN counting). The plates were incubated at 37 °C incubator for 3 days. Colonies on each plate are then counted and titers calculated. Geometric means of the counts in each group at the specified time points are presented. Error bars are standard error of mean. Filled bars, RB+LGG+EcN group; hatched bars, LGG+EcN group. PPD, post-partum day; PFD, post probiotic feeding starting day. Kruskal-Wallis rank sum test was used for comparisons. Different letters indicate significant differences between groups (n  = 10–18; p < 0.05), while shared letters indicate no significant difference.

To determine whether dietary RB can promote the growth and colonization of probiotic bacteria in Gn pigs, similar to the effects observed in mice^17^, the titers of LGG and EcN were determined on specified time points following 3 low oral doses (10^4^ CFU/dose) (Fig. 1). RB significantly increased the load of both gram-positive LGG and gram-negative EcN in the gut of Gn pigs. The RB fed pigs shed significantly higher counts of LGG (~10^4^ increases) starting from post-feeding day (PFD) 7 through the entire monitored period until PFD 30. Similarly, RB also significantly enhanced the shedding of EcN (~10^5^ increases) starting from PFD 2 through the entire monitored period until PFD 30. The peak titer for LGG shedding was 4 × 10^7^ CFU and for EcN was 3 × 10^8^ CFU. Together, these results showed that RB significantly enhanced the growth and colonization of the probiotic strains, with the effect on LGG growth manifested later and a slightly lower peak titer than that for EcN.

### RB increased pig body weight gain in LGG+EcN colonized pigs

Growth rate is an important indication for the gut and overall health of the host. To monitor the effect of RB on the growth rate of LGG+EcN colonized Gn pig, weekly body weight gains were compared between the LGG+EcN and the RB+LGG+ EcN pig groups (Table 2). RB increased the weekly body weight gain in the pigs on the 4^th^ and 5^th^ week after RB feeding started, as shown by the mean body weight gain of 0.75 versus 0.51 kg on the 4^th^ week, and 0.80 versus 0.42 kg on the 5^th^ week for the RB+LGG+EcN versus LGG+EcN group, respectively. These results suggest that RB can promote the growth of LGG+EcN colonized Gn pigs after 4 weeks of RB feeding, rendering the hosts more resistant to HRV infections and diarrhea.

### The combination of RB and LGG+EcN prevented epithelial damage from HRV challenge

**Figure 2:**
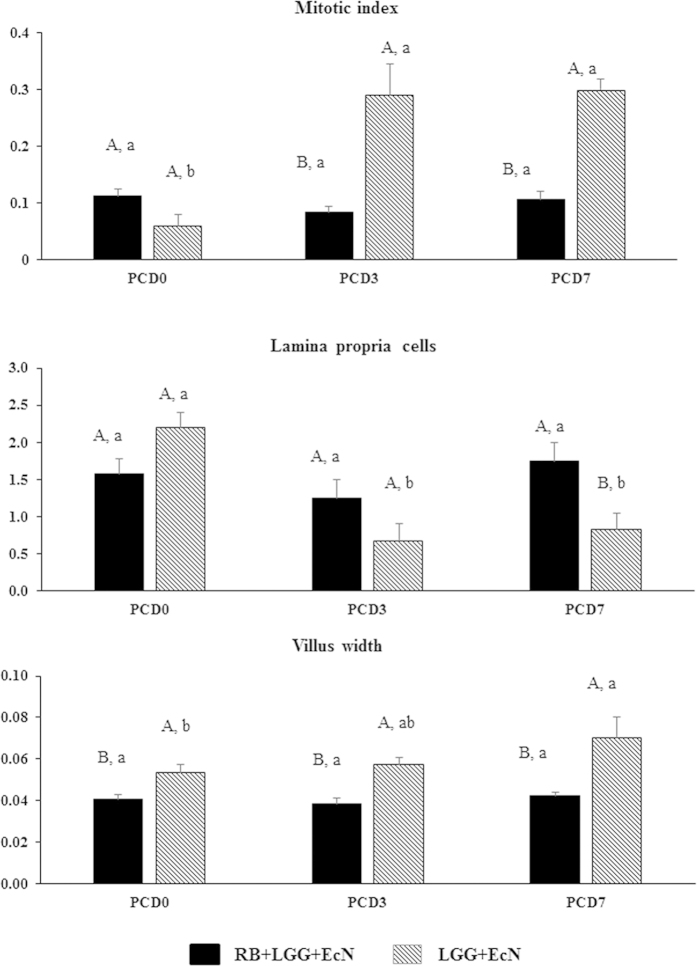
RB promoted intestinal epithelial health and maintained intestinal homeostasis during HRV infection in Gn pigs colonized with LGG and EcN. Selected data from Table 3 are represented in the figure. Kruskal-Wallis rank sum test was used for comparisons between different groups at the same time point (upper case letters) and between different time points for the same treatment group (lower case letters). Different letters indicate significant differences, while shared letters indicate no significant difference (n = 4–6; p < 0.05). For data and error bar descriptions, see Fig. 1 legend.

**Figure 3:**
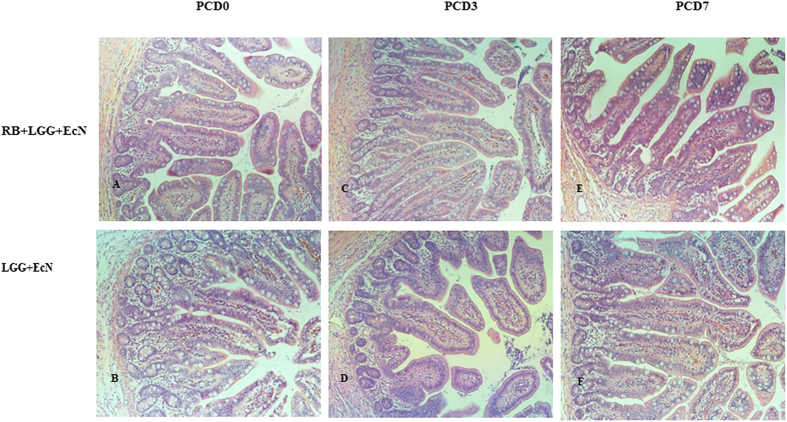
RB prevented the increase in villus width during HRV infection in Gn pigs colonized with LGG and EcN. Top panel are representative images for ileum sections of pigs in the RB+LGG+EcN group, whereas bottom panel shows the respresentative images for ileum sections of pigs in the LGG+EcN group. For each group, image for the non-infected pig on PCD 0 is shown on the left (**A**,**B**) the images for the HRV infected pigs on PCD 3 and PCD 7 are shown in the middle and on the right, respectively (**C**–**F**). On both PCD 3 and PCD 7, RB fed pigs have narrower or signficantly narrower villus width compared to the non-RB fed pigs, relfecting the reduced immune cell infiltration and edema generated during HRV induced inflammatory responses. Images were taken at 100× magnificantion. H & E stain.

To examine effects of RB on the health of intestinal epithelium of Gn pigs colonized with LGG and EcN, H&E stained slides of the distal ileum were evaluated and scored via light microscopy. The parameters and scores for the different treatment groups at different time points are shown in Table 3 and Fig. 2. Out of the all 7 parameters assessed, RB feeding significantly changed 3 parameters: mitotic index, villus width and the abundance of lamina propria cells (Fig. 2). Mitosis of intestinal crypt cells is increased to replace the damaged intestinal epithelial cells following HRV infection in Gn pigs. Dietary RB maintained the mitotic index in LGG and EcN colonized Gn pigs during HRV infection on PCD 3 and PCD 7, whereas LGG and EcN colonized Gn pigs without dietary RB feeding had significantly increased mitotic index on PCD 3 and PCD 7. Villus width is increased due to influx of immune cells and edema during rotavirus infection and inflammation. Dietary RB maintained the width of villus in ileum of LGG and EcN colonized Gn pigs during HRV infection on PCD 3 and PCD 7, whereas in the non-RB fed pigs, villus width increased significantly from PCD 0 to PCD 7 (Fig. 3). Additionally, RB maintained the abundance of lamina propria cells in LGG and EcN colonized Gn pigs during HRV infection, whereas non-RB fed pigs had significantly reduced lamina propria cells on PCD 3 and PCD 7. Furthermore, RB prevented the decrease in villus length during HRV infection from PCD 0 to PCD 3 and PCD 7, although these differences were not statistically significant.

When we compared the RB+LGG+EcN to the LGG+EcN pigs post HRV challenge at PCD 3 and 7 between the two treatment groups, RB fed pigs had significantly lower mitotic index and narrower villus width, but higher or significantly higher abundance of lamina propria cells and longer villus length, further supporting its protective effects against HRV induced ileum epithelial damage and inflammation, and its mucosal immune stimulatory effects. Therefore, RB not only protected against damage to intestinal epithelium, but also maintained the intestinal homeostasis (a balance of inflammation and immune response) in the face of HRV challenge.

### RB slightly reduced intestinal permeability during rotavirus infection

**Figure 4:**
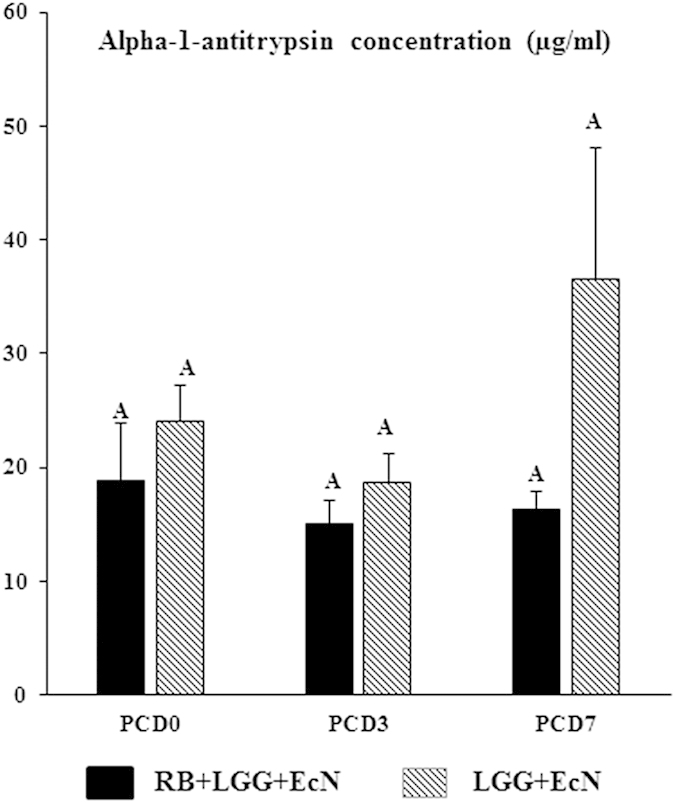
RB slightly reduced A1AT concentration (gut permeability marker) during HRV infection in Gn pigs colonized with LGG and EcN. LIC samples were collected in cryovials upon euthanasia and immediately frozen in liquid nitrogen until further analysis. Samples were diluted 3-fold before determination of the A1AT concentration with a commercial ELISA kit. Average value of duplicate for each sample was calculated first and then the means for all pigs in the same group at specific time point were calculated and are presented. Kruskal-Wallis rank sum test was used for comparisons and there were no statistically significant differences between treatment groups and among time points (n = 4–6), though there was a trend for lower A1AT concentrations in the RB+LGG+EcN group. For data and error bar descriptions, see Fig. 1 legend.

Serum level of A1AT is significantly elevated during the inflammatory response, and can be transported across the intestinal epithelial layer into intestinal lumen due to increased permeability of the intestinal epithelial barrier. A1AT is protease resistant and highly stable in the intestinal contents. Therefore, it has been used as a marker for inflammation and intestinal permeability^35^. In this study, we measured the A1AT level in LIC using ELISA (Fig. 4). RB maintained the level of A1AT in LIC during HRV infection on PCD 3 and PCD 7, whereas in non-RB fed pigs, A1AT level increased on PCD 7. However, these changes were not statistically significant due to high variability. When compared between RB and non-RB fed Gn pigs, RB fed pigs had a consistent trend of lower A1AT levels in LIC on PCD 0, PCD 3 and PCD 7. Therefore, RB may help maintain normal permeability in the ileum during the course of HRV infection.

### RB enhanced the innate immune response during HRV infection

**Figure 5:**
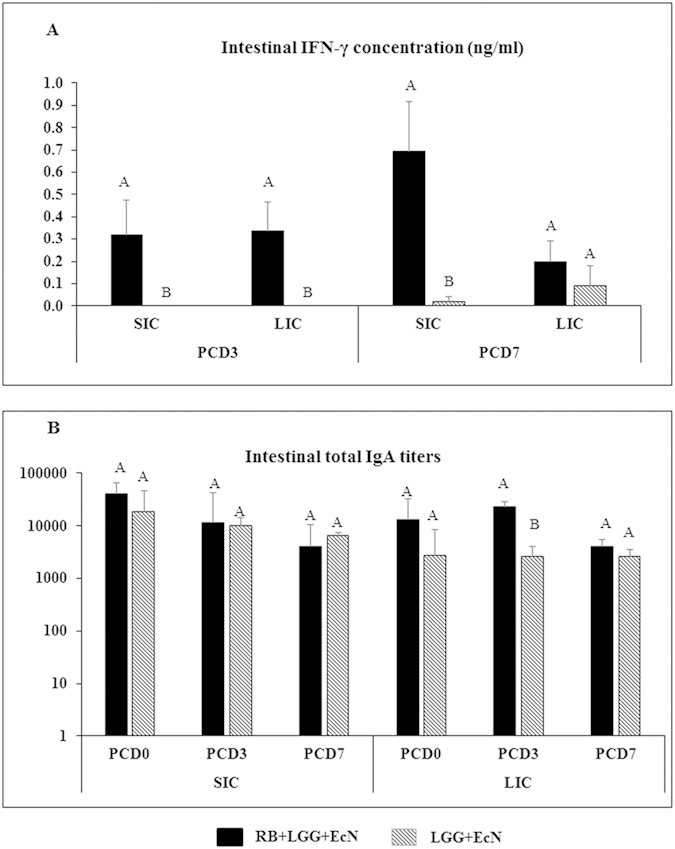
RB increased IFN-γ and total IgA levels in intestinal contents during HRV infection in Gn pigs colonized with LGG and EcN. SIC and LIC samples were collected upon euthanasia and stored in −20 °C until further analysis. Samples were diluted 2-fold before determination of the IFN-γ concentration with a commercial ELISA kit. Average value was calculated from duplicate for each sample and the means for all pigs in the same group at specific time point were calculated and are presented (**5A**). Kruskal-Wallis rank sum test was used for comparisons. Total IgA titers in intestinal contents were measured by ELISA and are presented as geometric mean titers for each treatment group (**5B**). ANOVA – Tukey test was used for comparisons. Different capital letters (**A**,**B**) indicate significant difference between the treatment groups for the same time point (n = 4–6; p < 0.05), while shared letters indicate no significant difference. For data and error bar descriptions, see Fig. 1 legend.

To evaluate the effects of RB plus LGG and EcN on innate immunity during HRV infection, we measured the intestinal IFN-γ concentration and total IgA and IgG titers in small and large intestinal contents using ELISA. RB+LGG+EcN group had significantly higher intestinal IFN-γ concentrations compared to the LGG+EcN group on PCD 3 in both SIC and LIC and on PCD 7 in SIC (Fig. 5A). IFN-γ concentrations did not differ between the two groups pre-challenge on PCD 0 (data not shown).

Compared to the LGG+EcN group, the RB+LGG+EcN group had significantly higher total IgA titers (9-fold; GMT 2580 versus 23171) in LIC on PCD 3 (Fig. 5B). No significant difference was observed for total IgA titers between the two groups on PCD 0; however, RB+LGG+EcN group had 5-fold higher IgA levels (GMT 2702 versus 13004) in LIC than the LGG+EcN group. No differences were detected in the total IgG titers at any time point between the two groups (data not shown). Together, these data suggest that RB strongly enhanced the innate protective immunity in LGG+EcN colonized pigs during HRV infection.

## Discussion

In this study we used the Gn pig model of HRV infection and diarrhea and demonstrated that dietary RB provided complete protection against HRV diarrhea in LGG and EcN colonized Gn pigs. The results also showed that dietary RB significantly enhanced the growth and colonization of both LGG and EcN in the intestine of Gn pigs, promoted body weight gain, protected against damage to intestinal epithelium while maintaining intestinal homeostasis (a balance of inflammation and immune response), maintained intestine permeability and enhanced the innate IFN-γ and IgA protective immunity during HRV infection. Together, these results demonstrated that the combination of RB and LGG and EcN can provide complete protection against HRV diarrhea and pointed to its potential mechanisms.

This is the first study that has tested the therapeutic effects of combined RB and probiotic LGG and EcN against HRV diarrhea. While RB, LGG, or EcN individually can confer varying degrees of protection against HRV diarrhea, this study showed that combining RB and initial colonization of the two diarrhea-reducing probiotics can achieve complete protection. We have previously showed that RB together with the oral AttHRV vaccine provided complete protection against HRV diarrhea in Gn pigs^18^. The complete protection against rotavirus diarrhea is significant as no rotavirus vaccines or antiviral drugs have shown such complete effectiveness, regardless of their specific mechanisms for reducing HRV diarrhea. RB’s effect on reducing diarrhea is not closely related to reducing HRV shedding. RB strongly enhanced the innate immunity as indicated by the increased intestinal IFN-γ and total IgA responses. Thus, this non-specific therapeutic effect against diarrhea suggests that the combination of RB and LGG+EcN could potentially be used to provide broad spectrum protection against diarrhea caused by other enteric pathogens and diarrhea of unknown etiology. Despite potential differences in the pathogenesis and molecular mechanisms of certain type of diarrhea between humans and pigs, it is expected that RB will enhance innate immunity and provide similar protection against diarrhea in humans. The optimal timing and dosage of RB and LGG and EcN may need to be further determined for each pathogen or type of diarrhea. Additionally, specific molecular mechanisms underlying this protection require further studies. Further clinical studies are currently ongoing to address these questions.

Despite their adjuvant effects on rotavirus vaccine and protection against rotavirus diarrhea^18^,^36^,^37^,^38^,^39^, neither RB alone nor LGG+EcN alone (Table 1) reduced HRV shedding in Gn pigs. LGG at both high dose and low dose did not reduce HRV shedding in our previous study^36^. Thus, RB and LGG+EcN seem to have similar effects on HRV pathogenesis, reducing diarrhea without impacting virus replication and shedding. Interestingly, RB significantly reduced HRV shedding in the LGG and EcN colonized pigs, as shown by significantly delayed onset of shedding, mean duration days and lower peak shedding titers (Table 1). The mechanism for this phenomenon is unclear, but may possibly be due to the synergistic effects of RB with LGG and EcN. LGG is normally found in gut microbiota of human infants and young children^40^. Therefore, RB, when used in humans who have already been colonized with LGG, is likely to be more effective in reducing HRV shedding than in germ-free pigs. LGG was found to significantly reduce rotavirus shedding in a conventional mouse model^38^. These results indicate that RB is promising to provide more effective protection against HRV diarrhea and significant reduction in rotavirus shedding in young children if their gut is colonized with LGG+EcN. This approach will be more effective, affordable and safer than probiotic therapy (reducing the potential risk of septicemia in immunocompromised children), especially for children in developing countries. Further pre-clinical studies in Gn pigs transplanted with human gut microbiota^39^ and human clinical trials of this novel therapeutic combination against HRV diarrhea and shedding are warranted.

In this study, dietary RB intake in Gn pigs increased the growth and colonization of both probiotic strains LGG and EcN up to 5 logs. This result supports previous findings that RB feeding can increase the abundance of the beneficial gut bacteria *Lactobacillus* spp. in mice^20^ and *Bifidobacterium* in adults^41^ and reduce the colonization and invasion of pathogenic *Salmonella enterica* in mice^20^. As LGG is a gram-positive bacterium in the Firmicutes phylum and EcN is a gram-negative bacterium in the Proteobacteria phylum, these results suggest that RB can promote the growth of a variety of probiotic strains. This is not surprising given the complex composition of RB^11^,^42^. However, the growth rate and abundance achieved by different probiotic strains with RB may be different. It is important to take these differences into consideration when using RB and probiotics clinically.

Rice bran enhanced the growth of Gn pigs, indicating increased nutrient absorption via the gut and overall health. It also promoted gut health by preventing epithelial damage (intestinal crypt cell mitosis) while maintaining the homeostasis of the mucosal immune system (maintained the number of lamina propria cells and villus width) during HRV infection in Gn pigs. During inflammation induced by HRV infection, intestinal permeability is increased, resulting in edema and diarrhea. Preventing the changes in gut permeability by RB may have contributed to its remarkable effects in preventing HRV induced diarrhea. Meanwhile, RB maintained the number of lamina propria cells, which are mainly lymphocytes (CD2+ and CD4+ T lymphocytes and sIgA secreting plasma cells)^43^, suggesting its ability to stimulate the intestinal mucosal immune system during HRV infection in LGG and EcN colonized pigs. Previous studies in mice^20^ and Gn pigs^18^ indicated that RB alone or together with probiotic bacteria such as *Lactobacillus spp* can increase the production of mucosal and systemic total IgA by plasma cells. Consistent with these findings, our result also showed RB significantly increased the intestinal total IgA titers during HRV infection. Thus, the increased lamina propria cells in the RB group pigs maybe due to its immune-stimulatory effects on the mucosal immune system. Together, the anti-inflammatory and immune modulatory effects of RB and LGG and EcN promoted intestinal epithelial health and homeostasis, contributing to an intact intestinal barrier that is resistant to HRV diarrhea.

It is not known which components or specific compounds of RB contributed to the HRV diarrhea reducing activities. However, heat-resistant amylase, protease and hemicellulase treated rice fiber, which has significantly lower contents of protein, lipids and carbohydrates, has been shown to be able to prevent diarrhea in dextran sodium sulfate (DSS) - induced experimental colitis mouse models^10^. This result suggests that the dietary fiber portion of RB, such as cellulose, hemicellulose and lignin, may also play important roles in decreasing diarrhea during inflammatory bowel disease. In fact, arabinoxylan, a dietary fiber from RB, significantly decreased the diarrhea score in irritable bowel syndrome adult patients through its anti-inflammatory and immune modulating activities^44^. RB components promoting probiotic bacteria growth and colonization are likely to vary depending on the specific bacterial species. However, heat-resistant amylase, protease and hemicellulase -treated dietary fiber was unable to increase the shedding of *Lactobacillus spp* and *Bifidobacterium*^10^, suggesting that carbohydrate or lipid components of RB could be the main prebiotics for LGG and EcN in this study. A recent study in mice found that a 10% RB oil diet significantly increased the occupation ratios of *Lactobacillales* group of bacteria in the gut microbiota^45^. Further studies are underway to identify the RB components that are responsible for its HRV diarrhea fighting properties and prebiotic properties.

Both RB and the probiotics are natural products, and have been demonstrated to have various health benefits for disease prevention and treatment in humans and animals. LGG has been shown to be safe in all age and health groups, even in immune-compromised individuals^46^. Given its natural colonization in the gastrointestinal systems, LGG has been studied extensively for its activities in treating gastrointestinal diseases and infections, such as diarrhea and enteric pathogens. However, this is the first study that showed the combined effects of RB and LGG+EcN in treating enteric pathogen infection and diseases. The results here indicated the synergistic effects of RB and LGG+EcN in preventing HRV diarrhea. Further preclinical studies in Gn pigs transplanted with human gut microbiota^39^ and human clinical studies are necessary to determine the optimal dosage and formulation for maximal safety and efficacy. In conclusion, the combination of RB and LGG+EcN may represent a novel, safe and highly effective therapeutic against diarrhea and infection caused by HRV, and potentially diarrhea caused by other enteric pathogens and etiologies in young children.

## Additional Information

**How to cite this article**: Yang, X. *et al.* High protective efficacy of rice bran against human rotavirus diarrhea via enhancing probiotic growth, gut barrier function, and innate immunity. *Sci. Rep.* **5**, 15004; doi: 10.1038/srep15004 (2015).

## Figures and Tables

**Table 1:** RB protected against HRV diarrhea and shedding in LGG and EcN colonized neonatal Gn pigs.

						Fecal virus shedding			
		Clinical signs				(ELISA)			(CCIF)
Treatments	N	% with diarrhea*,^a^	Mean days to onset**,§	Mean duration days**,†	Mean cumulative scores**,^b^	% shedding virus*	Mean days to onset**,§	Mean duration days**,†	Peak virus titer (FFU/ml)
RB+LGG+EcN	6	0^B^	N/A	0 (0)^B^	6.2 (0.5)^C^	100^A^	2.8 (0.3)^A^	5.2 (0.3)^C^	6.0 × 10^2 B^
LGG+EcN	6	50^B^	5.2 (1.3 c)^A^	0.7 (0.3)^B^	8.9 (0.6)^B^	100^A^	1.2 (0.2)^B^	6.8 (0.2)^A^	1.3 × 10^5 A^
RB only	5	20^B^	7.2 (0.8)^A^	0.2 (0.2)^B^	4.4 (1.6)^C^	100^A^	1.6 (0.2)^B^	6.2 (0.2)^B^	ND
Mock	9	100^A^	1.4 (0.2)^B^	5.6 (0.3)^A^	14.4 (1.0)^A^	100^A^	2.0 (0.3)^AB^	4.7 (0.7)^BC^	ND

^a^Pigs with daily fecal scores of ≥2 were considered diarrheic. Fecal consistency was scored as follows: 0, normal; 1, pasty; 2, semiliquid; and 3, liquid.

^b^Mean cumulative score calculation included all the pigs in each group.

^c^Standard error of the mean.

^§^In the groups where some but not all pigs had diarrhea or shedding, the onset of diarrhea or shedding for non-diarrheic/shed pigs were designated as 8 for calculating the mean days to onset.

^†^For days of diarrhea and virus shedding, if no diarrhea or virus shedding until the euthanasia day (postchallenge day 7), the duration days were recorded as 0.

^*^Fisher’s exact test was used for comparisons. Different letters indicate significant differences in protection rates among groups (n = 5–9; p < 0.05), while shared letters indicate no significant difference.

^**^Kruskal-Wallis rank sum test was used for comparisons. Different letters indicate significant differences in protection rates among groups (p < 0.05), while shared letters indicate no significant difference. ELISA, enzyme-linked immunosorbent assay; CCIF, cell culture immunofluorescent assay; FFU, fluorescence forming unit; N/A, not applicable; ND, not determined.

**Table 2:** RB enhanced the growth of Gn pigs from PPD27 to PPD33.

Treatment group	n	PPD13-19	PPD20-26	PPD27-33 (PCD0)	PPD34-40 (PCD7)
RB+LGG+EcN	12	0.58 (0.09)^A^	0.90 (0.08)^A^	0.75 (0.11)^A^	0.80 (0.08)^A^
LGG+EcN	16	0.54 (0.06)^A^	0.96 (0.05)^A^	0.51 (0.05)^B^	0.42 (0.17)^A^

RB (replacing 10% of total daily caloric intake) was added to the Gn pigs’ milk diet (ultra-high-temperature treated cow-milk) daily, starting at PPD 5 until the end of experiment. Weight gain over a specific period was calculated by subtracting the weight (in kilograms) at the beginning of a period from the weight at the end of the period. Mean weekly body weight gain of each treatment group is presented. Number in the parenthesis is standard error of mean. Kruskal-Wallis rank sum test was used for comparisons. Different letters indicate significant differences in weight changes between groups on the same time point (n = 3–16; p < 0.05), while shared letters indicate no significant difference. PPD, post-partum day; PCD, post challenge day.

**Table 3:** The effects of dietary RB on gut (ileum) epithelial health during HRV infection in Gn pigs colonized with LGG and EcN.

Treatments	Euthanasia day	n	Mitotic index	Villus length (mm)	Crypt depth (mm)	V:C	V:C score	Villus width (mm)	Lamina propria cells
RB+LGG+EcN	PCD 0	6	0.11 (0.01)	0.4 (0.02)	0.14 (0.01)	2.75 (0.14)	3.83 (0.17)	0.04 (0)^B^	1.58 (0.20)
	PCD 3	4	0.08 (0.01)^B^	0.4 (0.05)	0.13 (0.01)	2.98 (0.30)	3.5 (0.29)	0.04 (0)^B^	1.25 (0.25)
	PCD 7	6	0.11 (0.01)^B^	0.37 (0.02)	0.13 (0.01)	2.86 (0.17)	3.5 (0.22)	0.04 (0)^B^	1.75 (0.25)^A^
LGG+EcN	PCD 0	5	0.06 (0.02)^b^	0.37 (0.01)	0.14 (0.01)	2.73 (0.18)	3.6 (0.24)	0.05 (0)^Ab^	2.2 (0.20)^a^
	PCD 3	6	0.29 (0.06)^Aa^	0.33 (0.03)	0.14 (0.00)	2.41 (0.22)	4.00 (0)	0.06 (0)^Ab^	0.67 (0.25)^b^
	PCD 7	6	0.30 (0.02)^Aa^	0.30 (0.03)	0.13 (0.01)	2.75 (0.23)	3.67 (0.21)	0.07 (0.01)^Aa.^	0.83 (0.21)^Bb^

After euthanasia, H & E stained sections of ileum from each pig were prepared and read by a veterinary pathologist blinded to the identity of the animals. Mean values for each parameter is presented. Number in the parenthesis is standard error of mean. Kruskal-Wallis rank sum test was used for comparisons. Different upper case letters indicate significant differences between groups at the same time point; different lower case letters indicate significant differences among the time points within the same group (n = 4–6; p < 0.05), while shared letters or no letters indicate no significant difference.
